# MIG1 Glucose Repression in Metabolic Processes of *Saccharomyces cerevisiae:* Genetics to Metabolic Engineering

**Published:** 2019

**Authors:** Iraj Alipourfard, Nelly Datukishvili, Salar Bakhtiyari, Karimeh Haghani, Laura Di Renzo, Renata Costa de Miranda, David Mikeladze

**Affiliations:** 1. Institute of Chemical Biology, Faculty of Natural Sciences and Engineering, Ilia State University, Tbilisi, Georgia; 2. Center of Pharmaceutical Sciences, Faculty of Life Sciences, University of Vienna, Vienna, Austria; 3. Department of Clinical Biochemistry, Faculty of Medicine, Ilam University of Medical Sciences, Ilam, Iran; 4. Section of Clinical Nutrition and Nutrigenomics, Department of Biomedicine and Prevention, University of Rome Tor Vergata, Rome, Italy; 5. Faculty of Applied Medical-Surgical Sciences, University of Rome Tor Vergata, Rome, Italy; 6. CAPES Foundation, Ministry of Education of Brazil, Brasília, Brazil

**Keywords:** *Saccharomyces cerevisiae*, Homologous recombination, Metabolic pathways, Yeasts

## Abstract

**Background:**

Although *Saccharomyces cerevisiae* has several industrial applications, there are still fundamental problems associated with sequential use of carbon sources. As such, glucose repression effect can direct metabolism of yeast to preferably anaerobic conditions. This leads to higher ethanol production and less efficient production of recombinant products. The general glucose repression system is constituted by *MIG1*, TUP1 and SSN6 factors. The role of *MIG1* is known in glucose repression but the evaluation of effects on aerobic/anaerobic metabolism by deletion of *MIG1* and constructing an optimal strain brand remains unclear and an objective to be explored.

**Methods:**

To find the impact of *MIG1* in induction of glucose-repression, the Mig1 disruptant strain (Δ*MIG1*) was produced for comparing with its congenic wild-type strain (2805). The analysis approached for changes in the rate of glucose consumption, biomass yield, cell protein contents, ethanol and intermediate metabolites production. The *MIG1* disruptant strain exhibited 25% glucose utilization, 12% biomass growth rate and 22% protein content over the wild type. The shift to respiratory pathway has been demonstrated by 122.86 and 40% increase of glycerol and pyruvate production, respectively as oxidative metabolites, while the reduction of fermentative metabolites such as acetate 35.48 and ethanol 24%.

**Results:**

Results suggest that Δ*MIG1* compared to the wild-type strain can significantly present less effects of glucose repression.

**Conclusion:**

The constructed strain has more efficient growth in aerobic cultivations and it can be a potential host for biotechnological recombinant yields and industrial interests.

## Introduction

The yeast *Saccharomyces cerevisiae* (*S. cerevisiae*) with safety of handling and capable of genetic transfer system is a favorite organism for protein production. The growth of *S. cerevisiae* as a potential eukaryotic host requires utilizing carbon resources in optimal process. Industrial carbon sources usage by yeast slow down when glucose and fructose are present in culture media. This is commonly called carbon catabolite repression (referred to as glucose repression) 1,2.

The presence of glucose in industrial cultivation media has a negative impact upon the metabolism of other sugars. Glucose repression reduces the transcripttion rate of repressible genes, and is the most investigated mechanism of glucose control. So, it is of importance to develop strains in which glucose repression is relieved 3. Therefore, all sugars should be utilized, preferably simultaneously, to achieve optimal economic yield 4. Invertase (EC 3.2.1.26), the sucrose-hydrolyzing enzyme, is expressed under control of glucose repression. In *S. cerevisiae*, six *SUC* genes (*SUC1* to *SUC5* and *SUC7*) are responsible to encode Invertase of which *SUC2* is the most common one 2. Invertase is encoded by *SUC2* including two forms, secreted and cytoplasmic 5.

The secreted form of invertase is glycosylated and located in periplasmic space, digesting extracellular sucrose to glucose and fructose, both of which can be transported into the cell 6. The cytoplasmic form is not glycosylated, and acts physiologically to cleave sucrose, transported across the plasma membrane 7,8.

*SUC2* gene repression following glucose uptake constitutes a regulatory cascade through a large number of regulatory elements 2. Mig1p, a zinc finger DNA-binding protein, is the main involved component that mediates glucose induced repression of sugars. Inactivation of *MIG1* gene derepresses the expression of invertase encoded by *SUC2* 9. This protein resembles the mammalian Egr and Wilms’ tumor proteins, the CreA repressor in *Aspergillus nidulans* and the Migl repressor in *Kluyveromyces lactis* 4,5.

The *MIG1* promoter has been reported to be auto-regulated 6. Mig1p prevents transcription of *SUC2* through the mechanism that mediates the binding of the general repressor Ssn6p-Tup1p complex to the regulatory part of *SUC2*. Snf1 kinase regulates glucose derepression of genes required for utilization of alternative carbon sources. The main mechanism is through glucose dependent dephosphorylation of transcriptional repressor protein Mig1. One important strategy to reveal the mechanism of Mig1-mediated repression is to investigate the physiological consequences of *MIG1* deletion/disruption and/or *MIG1* over expression 3. No absolute evidence could well explain the physiological changes by Mig1-redundant protein in a ΔMIGl mutant, *i.e*. a protein that is similar to Miglp and can partly be a substitute. A second repressor, Mig2p, which is 71% identical to Miglp, as well as a related protein, YerO28, have been identified. In microarray approached combinatorial control, *MIG1* and MIG2 repress a largely overlapping set of genes but MIG3 does not seem to overlap in function with *MIG1* and MIG2. Instead, MIG3 down regulates the SIR2 gene responsible for gene silencing and the control of aging 8. There are two approaches to silence *MIG1* gene of which one is usually accomplished by disruption and substitution of the target gene with a selecting marker introduced by homologous recombination. The second alternative approach, which has been applied and researched in plant, is antisense gene expression 9,10.

In this study, the purpose was to apply the first approach to disrupt chromosomal *MIG1* gene through homologous recombination with reconstructed plasmid containing inserted N and C terminals of *MIG1* gene coupled with URA3 selecting marker enabling Δ*MIG1* mutants to be screened. Disruption and replacement of *MIG1* can derepress promoters of genes which are repressed by glucose. Prototrophic *MIG1* disruptant (Δ*MIG1*) as well as its congenic wild-type strain (2805) were analyzed for expected physiological changes in peripheral metabolism (batch cultivations on sugar mixtures) and central metabolism. During the cultivations, an attempt was made to monitor the parameters of glucose consumption, biomass production, intracellular protein content and patterns of metabolite production to understand how the effect of *MIG1* removal can shift to oxidative pathway of metabolism. The strain can be optimized as a host for further processing of recombinant production with advantages of being eukaryotic.

## Materials and Methods

### Preparation of YPD broth culture to grow the yeast

*S. cerevisiae* wild type strain 2805 was cultured on solid or liquid Yeast Peptone Dextrose (YPD), while carbohydrates were supplied at concentrations of 20 *g/L* 11. For physiological experiments, a medium, previously described, was used with minor modifications 12. Medium for batch cultivations consisting of glucose (28 *mM*), sucrose (58 *mM*), (NH_4_) 2SO_4_ (7.5 *g/L*), KH_2_PO_4_ (3.5 *g/L*), MgSO_4_· 7H2O (2.7 *g/L*), trace metal solution (20 *ml/L*), vitamin solution (2 *ml/L*), and antifoam 289 (50 *ml/L*) were used. The trace metal solution was composed of EDTA (3 *g/L*), CaCl_2_·2H_2_O (0.9 *g/L*), ZnSO_4_·7H_2_O (0.9 *g/L*), FeSO_4_·7H_2_O (0.6 *g/L*), H_3_BO_3_ (0.2 *g/L*), MnCl_2_·2H_2_O (155.5 *mg/L*), Na_2_MoO_4_·2H_2_O (80 *mg/L*), CoCl_2_·2H_2_O (60 *mg/L*), CuSO_4_·6H_2_O (60 *mg/L*), and KI (20 *mg/L*), adjusted to pH=4.0 with NaOH. The vitamin solution was composed of *d*-biotin (50 *mg/L*), *p*-aminobenzoic acid (200 *mg/L*), nicotinic acid (1.0 *g/L*), calcium pantothenate (1.0 *g/L*), pyridoxine HCl (1.0 *g/L*), thiamine, HCl (1.0 *g/L*), and *m*-inositol (25 *g/L*), adjusted to pH=6.5 with HCl 3. Sugars were autoclaved separately, and the vitamin solution was sterilized by filtration and added after autoclaving. To prevent bacterial growth antibiotics penicillin G, streptomycin and chloramphenicol were added to media in concentration 100, 100 and 50 *μgr/L*, respectively along with adding 2% agar which can provide YPD solid media. The preparation of the inoculum and the conditions of batch cultivation were according to the described experiment 13: a colony taken from YPD agar plate inoculated in 4 baffled Erlenmeyer shake flasks containing 250 *ml* YPD broth at 28*°C* and 100 *rpm* for 48 *hr*. The temperature was kept fixed and the pH was controlled within the range 4.9–5.0 by automatic addition of 4 *M* NaOH. The air flow was set to 1 *vvm* (4.0 *L/min*), and the off-gas was led through a condenser. After incubation period, media were centrifuged and washed by normal saline within 50 *ml* tubes in 3000 *rpm* for 10 *min*.

### Preparation and analysis of DNA

Total yeast DNA was extracted as described by Ausubel *et al* 14. To check purification of extracted DNA, quantitative control was performed by spectrophotometric method 15 and quality of DNA was checked by accuracy and sharpness of related bands in gel electrophoresis. For restriction analysis and gel electrophoresis, experiments standard protocols were followed 16. For the purification of DNA fragments used for cloning experiments, the desired fragments were separated on 0.7% agarose gels, excised and recovered from agarose, using BIOTRAP BT1000 (Schleicher & Schuell, Duren, Germany).

### Oligonucleotide primers and PCR analysis

Both forward and reverse primers provided by Oligo and Blast software for each of N-fragment (before starting codon of *MIG1*) and C-fragment (after stop codon of *MIG1*) included restriction sites for EcoRI and BamHI. Primers were made by Clontech Company according to the N- and C-terminal sequences of *MIG1* gene. PCR amplification of N and C terminal fragments was performed by thermocycler according to instructions followed: initial single denaturation at 94*°C* for 5 *min*, 30 cycles of denaturation at 94 for 30 *s*, annealing at 45*°C* for 45 *s*, extension at 72*°C* for 1 *min* and final extension at 72*°C* for 5 *min*. The correct amplification of PCR products (N-fragment of 400 *bp*, C-fragment of 600 *bp*) was confirmed by gel electrophoresis. In table 1, the oligonucleotide primers used in PCR analysis are shown.

**Table 1.** Oligonucleotide primers used in PCR analysis

**Primer**	**Enzyme**	**Restriction site**
**F1**	XbaI	CCC**TCTAGA**GAATACTGAACGCCATAG
**R1**	BamHI	CCC**GGATCC**TAGCGTACTTATTATGTG
**F2**	BamHI	CCC**CGGATC**TGCCCTTTTTCTACTTAT
**R2**	EcoRI	CCC**GAATTC**CAACATTCCGGAATCTGAA

### Cloning of N and C fragments and transformation to Escherichia coli (E. coli)

The amplified N and C fragments were cleaned up by PCR product purification kit (Biogen Company). The purified products enabled restricted digestion of fragments on suitable restriction sites which existed within the primers. Namely, N fragments were digested with EcoRI and BamHI enzymes and C fragments were digested with XbaI and BamHI enzymes. The restriction digestion reactions were carried out for overnight at 37*°C*. The enzymes were inactivated in 10 *min* at 65*°C*. After gel electrophoresis of digested fragments, 400 and 600 *bp* DNA bands corresponding to N-and C-fragments, respectively, were extracted from gel 16. The gel purified N- and C-fragments were inserted into plasmid pBluescript II SK (Stratagene) using Fermentase DNA cloning kit (Thermo Fisher Scientific Company). The resulted constructed plasmid pMIG1 consists of BamHI as midline restriction site between inserted N- and C-fragments. Furthermore, plasmids were transferred to *Escherichia coli (E. coli)* DH5α (Bethesda Research Laboratories). *E. coli* was transformed by electroporation using a Gene Pulser (Pulse Controller, Bio-Rad, Richmond, CA, USA) according to the manufacturer’s instructions. The growth of transformants in LB culture media containing ampicillin (100 *mg/ml*) and X-α-gal (as screening agent) and IPTG (as galactose metabolism inducing factor) allowed selection of white colonies consisting of pMIG1 plasmid. The recombinant pMIG1 plasmid consisted of plasmid pBluescript, N- and C-fragments digested with BanHI and inserted with selection marker, URA3, as described by Klein *et al* 17.

### Transformation to yeast by pMIG1 plasmid

The wild strain *S. cerevisiae* was transformed by PEG/Li acetate method 16 to replace to produce *MIG1* disrupted strains. URA3 enables recombinant yeast to synthesize required uracil for growing in uracil-lacked medium (SC-URA).

### Determining cell biomass

Produced biomass of wild and *MIG1* disrupted strains was determined by using optical densitometry method. The biomass yield was obtained from the slopes of the plots of biomass and the amount of consumed sugar during exponential growth phase.

### Analysis of sugars and produced metabolites

To measure sugars and extracellular metabolites, cultivation medium was sampled, immediately filtered through a 0.45 *mm* pore size cellulose acetate filter. Glucose, galactose, maltose, ethanol, glycerol, acetate and pyruvate were separated on an Aminex HPX-87H ion exclusion column HPLC and were detected refractometrically or spectrophotometrically 13.

### Measurement of cell protein content

For the determination of cell protein content, a sample was isolated in ice beaker, centrifuged and washed three times with 5 *ml* of 0.9% (*w:w*) NaCl and resuspended in 1 *ml* of water, before storage at −80*°C*. Then, the total protein content was measured according to the Biuret method 12.

## Results

### Construction of disrupted mutant yeast

**Figure 1.**
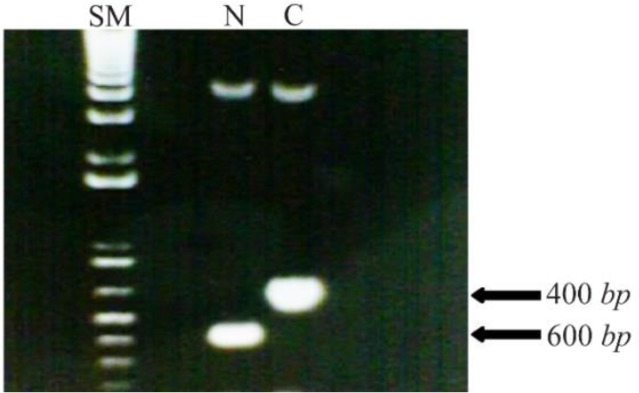
Amplified N fragment (400 *bp*) and C fragment (600 *bp*) of MIG1 chromosomal gene of Saccharomyces Cerevisiae next to the size marker. The size marker (SM) contains of bands for each equals to 100 *bp.*

The haploid wild type strain 2805 was selected to test the feasibility of suppressing effect of *MIG1* by gene disruption and to simplify direct isogenic comparison of *MIG1* expressing strains with that of *MIG1* disrupted mutants. The confirmation of amplification of N and C fragments of *MIG1* gene sequence with 400 and 600 *bp*, respectively, were compared to size marker (Figure 1). Insertion of N and C fragments in pBlueskript II plasmid and transformation to *E. coli* DH5a leading to antibiotic resistance attributed to the bacterial aminoglycoside phosphotransferase-encoding *APT2* gene which is inserted between the *PGK* promoter and terminator.

After replica plating to X-α-Gal medium under inducing conditions with galactose and glycerol, transformed bacterial strains were isolated (data not shown). Upon transformation of strain 2805 with pBluescript II integrated with N and C fragments of *MIG1* sequence interrupted with URA3, several clones of yeast able to grow in SC-URA were isolated.

### Metabolic assessment

For batch cultivations on glucose, each of the *Saccharomyces* strains 2805 and Δ*MIG1* were grown on 40.0 *g/L* of glucose 3. The results were from the study of aerobic metabolism under defined conditions of glucose control. Each experiment was repeated at least three times. The achieved results were analyzed by using SPSS 21.0 (SPSS, Chicago, IL) software. All normally distributed continuous variables were demonstrated as mean.

### Glucose consumption and growth rate

**Figure 2.**
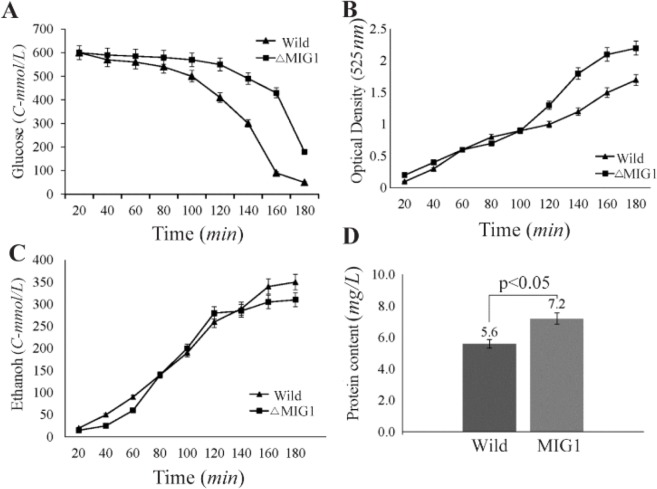
Concentration of A) glucose, B) cell mass, C) ethanol, and D) cell protein in batch cultivations of wild strains 2805(■) and ΔMIG1 (MIG1 disrupted mutant) (▲) on a medium with glucose control conditions. The data achieved of three independent experiences and calculated mean values have been demonstrated for each amount on the related graph, (n=3), p<0.05, significantly different characteristics of mutant strain from respective wild strain.

Glucose consumption in Δ*MIG1* mutant was proceeded to wild 2805 strain by 25.0% within several batch and lag phase measurements (Figure 2A). The maximum specific growth rate of 2805 and of Δ*MIG1*, calculated from nine simultaneous optical density measurements demonstrated 0.39 and 0.43 *h*^−1^, respectively. Thus, *MIG1* disruption led to 12.0% average increase of specific growth rate on glucose (Figure 2B).

### Ethanol production and cell protein content

In batch cultivation, the rate of carbon flux directed to ethanol production in Δ*MIG1* strain was reduced about 24.0% (*C-mmol/L*) of lag phase cultivation (Figure 2C). The less formation of ethanol and related anaerobic metabolites at a higher rate with Δ*MIG1* than with 2805 demonstrates considerable shifted metabolism from respiro-fermentative to respiratory pathway. Intracellular protein content accounted for both strains showed 22.0% (*w:w*) difference to significant increase in Δ*MIG1* compared with 2805 (Figure 2D).

### Glycerol, pyruvate and acetate formation

**Figure 3.**
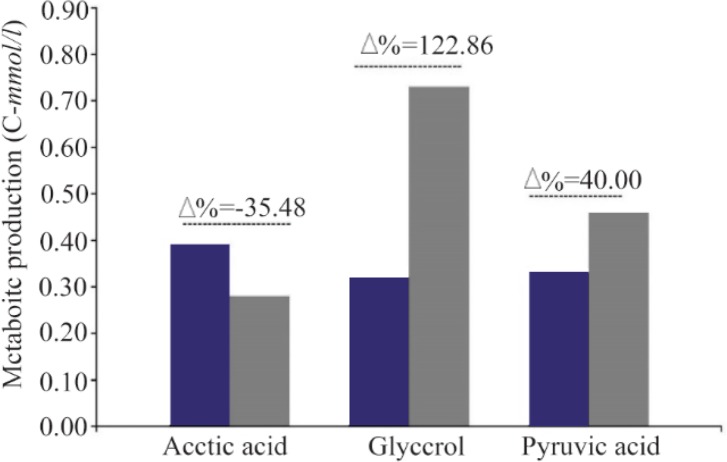
Different production ratio (Percent variation Δ%) of metabolite parameter (Acetic acid, Glycerol, Pyruvic acid) in batch cultivations of wild strains 2805 (■) and ΔMIG1(MIG1 disrupted mutant) (■) on a medium with glucose control conditions.

The carbon fluxes have been increased to respiratory metabolism pathway. It is indicated by more remarkable formation of oxidative metabolites markers, *i.e* glycerol and pyruvate which have been produced 122.86 (*C-mmol/L*) and 40.0% (*C-mmol/L*) in Δ*MIG1* prominent than strain 2805, respectively. Conversely, the decrease of fermentative metabolite of acetate by 34.58% (*C-mmol/L*) has been observed. The differences between the rate of metabolite production within cultivation period have been calculated and expressed as percentage (Δ%) (Figure 3). The comparison between all continuous variables was performed by using one-way analysis of variance (ANOVA). If there were statistically significant differences, then Tukey’s Post-Hoc test was applied. The values of p<0.05 were considered statistically significant.

## Discussion

Yeast has been considered as a perfect microorganism for biological studies and protein recombinant production. Safety of handling and synthesized recombinant products also capable genetic transfer systems make this organism to be widely used as a host. In yeast cells, unification of transformed DNA and chromosomes potentially occur through homologous recombination process leading to expression of different modified eukaryotic proteins.

The major types of yeast being used in biotechnology as gene expression tools are *S. cerevisiae, Hansenula polymorpha,* and *Kluyveromyces lactis*. For several advantages, *S. cerevisiae* is well selected for heterologous protein production. In this research, the chromosomal metabolic gene, *MIG1*, disrupted by homologous recombination through antisense technique using upward and downward, N- and C-, fragments of *MIG1* gene was applied to study the metabolic effects on yeast. Lateral fragments, N and C, after amplification were inserted in yeast specified vector, pTcURA3 alongside metabolic selection marker URA3. After transformation to yeast, *MIG1* gene would be replaced with URA3 marker. It can be happened by recombination between its lateral N and C fragments and resembles sequences already inserted into the vector lateral to URA3 marker. This transformation enables the yeast to synthesize required nutrient uracil. In fact, through this method, *MIG1* disrupted strain can be screened by growth on uracil negative medium. Then, further analyses of central and peripheral metabolic pathway have been done to approach variations between mutant and intact wild strain.

The glucose accessibility is the most important environmental factor to regulate oxidation and fermentation metabolic pathways of *Saccharomyces i.e*., glucose concentration in batch inhibits the utilization of other carbon resources, the catabolic inhibition 18. Hexokinase PII starts glucose inhibition cascade 18 and Snf1p as a signal transductor phosphorylates mig1p (regulatory zinc finger protein of *MIG1* gene) which makes inhibitory complex with Ssn6 (cyc8)-tup1 components (Carlson, 1987) and binds to promoter of glucose controlled genes. The *MIG1* gene was cloned in 1990 for the first time 4. Deletion of *MIG1* demonstrates a greater impact on peripheral functions than on central metabolism.

There are recent evidences which persist on the role of Hexokinase 2 (Hxk2) in regulation of DNA-binding repressor proteins of Mig1 protein and glucose repression signal in nucleus. Hxk2 acts as an intracellular glucose sensor that operates by changing its conformation in response to cytoplasmic glucose levels and regulates dephosphorylation of Mig1 19,20. In the present study, Δ*MIG1* strain 2805 could utilize more glucose in comparison to the wild strain within the first 12 *hr* to reach to the zero point. When the glucose concentration is high, Snf1p is inactivated and non-phosphorylated mig1p would remain in nucleus and represses transcription of dependent genes. Conversely, in low concentration of cultivation glucose, phosphorylated MIG1p can migrate to cytoplasm and leads to removal of repression status. The deletion of *MIG1* can eliminate glucose repression partially from peripheral functions, rather than completely from central metabolic functions. The outcome of this deletion has been considered significant in industry because glucose is the most useful sugar among the carbon resources for yeast in industry process. This can delay uptake of other sugars resulting in elongation of production process. Therefore, an effective alleviation of glucose control would help to achieve a better process economy for the cultivation of baker’s yeast, alcohol fermentation, bread-making and recombinant protein production 17. In some studies, it has been proved that disruption of *MIG1* cannot remove the entire glucose repression effect from galactose, maltose and sucrose metabolism. Therefore, substantial *MIG1*-independent glucose control mechanisms exist for the GAL, MAL and *SUC* systems 21. On the other hand, glucose through MIG1 regulation process has several repression effects on the expression of GAL family genes like *GAL2*, *GAL3*, *GAL4*, and *GAL80*. They are involved in the metabolism of galactose and highly affected by *MIG1* disruption 22,23. Imp2p is a *MIG1* related regulator of *GAL* genes and has the positive effect on glucose derepression of the maltose, galactose and raffinose utilization pathways and in resistance to thermal, oxidative or osmotic stress of *S. cerevisiae* 23. The finding that CAT8 was derepressed in a ΔMIGl strain, whereas Cat8-controlled FBPl, PCKl and ZCLl were not, gives a clear hint of the existence of other repressors such as MIG2 or a post-translational modification of an effector such as the phosphorylation of the derepressor Cat8 16.

The study of the effect of simultaneous deletion of *MIG1*/*MIG2* genes on physiology of *Saccharomyces* exhibited that glucose control of maltose and sucrose metabolism was derepressed in disrupted *MIG1/MIG2* 3,24. However, the lag phase of galactose cultivation diminished and further deletion of MIG2 could not affect glucose dependent metabolism of galactose. The *MIG1* gene was silenced by antisense *MIG1* expression 10. The results of evaluation of glucose consumption, cell biomass production and intracellular protein contents by Optical Densitometry (OD) method and Invertase enzymatic activity assays demonstrated the more flexible growth of Δ*MIG1* strains under the sufficient amount of oxygen and glucose. This is evidential for removal of glucose repression effects and impressive shift to more oxidative metabolism in comparison to wild strains. Yeast cell cultivations are effectively stressed by chemical metabolites produced within growth phase namely acids and high percentage of ethanol which can suppress the yeast growth. Deletion of *MIG1* can switch fermentative metabolism to aerobic pathway. It results in the production of less fermentative products like ethanol and acetate. Meanwhile, the promotion of Krebs cycle leads to more acidic derivative products such as glycerol and pyruvate. The data from the current research approved the similar concept 12,25. However, the increase of glycerol production in mutant strain is much prominent than what has been reported in similar studies 10,13. Such modifications in peripheral and central metabolic functions could satisfy industrial interests which attempt to have optimal engineered strains for recombinant production.

## Conclusion

This study showed the amount of ethanol measured by HPLC method in mutant and wild strain cultivations which determined less production of Δ*MIG1*. Enhancement of biomass production in Δ*MIG1* strains can lead to higher amount of intracellular protein content and carbohydrates. In the current research, results of strain Δ*MIG1* 2805 could confirm similar achievements rather than wild types. As to peripheral functions, efforts have already been exerted in production of partially derepressed galactose, maltose and sucrose metabolisms. For central functions, industrial interest could promote attempts in the metabolic engineering of strains that exhibit higher specific growth rates for baker’s yeast or recombinant protein production, or of distiller’s yeast strains that give higher ethanol yields.
